# Development of an Interpretable Deep Learning Model for Pathological Tumor Response Assessment After Neoadjuvant Therapy

**DOI:** 10.1186/s12575-024-00234-5

**Published:** 2024-04-17

**Authors:** Yichen Wang, Wenhua Zhang, Lijun Chen, Jun Xie, Xuebin Zheng, Yan Jin, Qiang Zheng, Qianqian Xue, Bin Li, Chuan He, Haiquan Chen, Yuan Li

**Affiliations:** 1 Department of Pathology, Fudan University Shanghai Cancer Center, Shanghai, China 200032; 2 Department of Oncology, Shanghai Medical College, Fudan University, Shanghai, China 200032; 3 Shanghai Aitrox Technology Corporation Limited, Shanghai, China; 4 Department of Future Technology, Shanghai University, Shanghai, China; 5 Department of Thoracic Surgery, Fudan University Shanghai Cancer Center, Shanghai, China

**Keywords:** Pathological Tumor Response, Immunochemotherapy, Esophageal Squamous Carcinoma, Knowledge Distillation, Semi-supervised Learning

## Abstract

**Background:**

Neoadjuvant therapy followed by surgery has become the standard of care for locally advanced esophageal squamous cell carcinoma (ESCC) and accurate pathological response assessment is critical to assess the therapeutic efficacy. However, it can be laborious and inconsistency between different observers may occur. Hence, we aim to develop an interpretable deep-learning model for efficient pathological response assessment following neoadjuvant therapy in ESCC.

**Methods:**

This retrospective study analyzed 337 ESCC resection specimens from 2020–2021 at the Pudong-Branch (Cohort 1) and 114 from 2021–2022 at the Puxi-Branch (External Cohort 2) of Fudan University Shanghai Cancer Center. Whole slide images (WSIs) from these two cohorts were generated using different scanning machines to test the ability of the model in handling color variations. Four pathologists independently assessed the pathological response. The senior pathologists annotated tumor beds and residual tumor percentages on WSIs to determine consensus labels. Furthermore, 1850 image patches were randomly extracted from Cohort 1 WSIs and binarily classified for tumor viability. A deep-learning model employing knowledge distillation was developed to automatically classify positive patches for each WSI and estimate the viable residual tumor percentages. Spatial heatmaps were output for model explanations and visualizations.

**Results:**

The approach achieved high concordance with pathologist consensus, with an R^2 of 0.8437, a RAcc_0.1 of 0.7586, a RAcc_0.3 of 0.9885, which were comparable to two senior pathologists (R^2 of 0.9202/0.9619, RAcc_0.1 of 8506/0.9425, RAcc_0.3 of 1.000/1.000) and surpassing two junior pathologists (R^2 of 0.5592/0.5474, RAcc_0.1 of 0.5287/0.5287, RAcc_0.3 of 0.9080/0.9310). Visualizations enabled the localization of residual viable tumor to augment microscopic assessment.

**Conclusion:**

This work illustrates deep learning's potential for assisting pathological response assessment. Spatial heatmaps and patch examples provide intuitive explanations of model predictions, engendering clinical trust and adoption (Code and data will be available at https://github.com/WinnieLaugh/ESCC_Percentage once the paper has been conditionally accepted). Integrating interpretable computational pathology could help enhance the efficiency and consistency of tumor response assessment and empower precise oncology treatment decisions.

**Supplementary Information:**

The online version contains supplementary material available at 10.1186/s12575-024-00234-5.

## Introduction

Esophageal carcinoma persists as the 7th leading cause of cancer mortality worldwide, with esophageal squamous cell carcinoma (ESCC) being the predominant subtype, associated with substantial morbidity and mortality in China [1, 2]. While preoperative chemoradiotherapy followed by surgery has become the standard of care for locally advanced ESCC [2–4], the prognosis remains dismal for many patients. Recently, the advent of neoadjuvant immunotherapy has demonstrated remarkable efficacy and favorable safety across numerous malignancies [5]. Consequently, there has been growing enthusiasm for leveraging neoadjuvant chemoimmunotherapy regimens to improve outcomes in ESCC [4, 6–8]. Determining optimal integration of immunotherapies with conventional neoadjuvant strategies represents an urgent unmet need with profound implications for enhancing the historically poor prognosis of this deadly disease.

Accurate pathological response assessment is critical to assess therapeutic efficacy and determine the optimal post-operation regimens for cancer patients. Currently, tumor regression grade (TRG), pathological complete response (pCR) and major pathological response (MPR) [9–11] serve as the predominant metrics for pathological response assessment, quantifying the percentage of viable residual tumor cells in resected specimens following neoadjuvant therapy [9, 12]. Both pCR and MPR, defined by no viable residual tumor or no more than 10% viable residual tumor, are considered as important surrogate trial endpoints [13, 14]. However, pathological assessments can be labor-intensive and the results among different observers may exhibit inconsistency.

Our study therefore aimed to develop and validate a novel deep learning-based approach to serve as a scalable tool to substantially help enhance the pathological response assessment after neoadjuvant therapy, which could help reduce the workload and standardize the assessment results, unlocking the full potential of personalized post-operation treatment, especially in high-risk areas.

Recent advances in artificial intelligence have sparked growing interest in applying deep learning approaches to transform cancer diagnostics and clinical practice [15, 16]. While deep learning models have shown promise for classification and segmentation tasks using whole slide images [17–22], new challenges have emerged for assessing tumor regression grade. First, convolutional neural networks struggle to jointly extract local tissue details and global slide-level features due to the vast scale of whole slide images [23, 24]. Second, time-intensive manual annotation makes large supervised training datasets infeasible, prompting weak supervision methods that leverage slide-level labels to infer patch-level information [25, 26]. However, this patch-level inference lacks robustness when slide-level labels contain noise. This is often the case for pathologist-derived tumor percentage estimates serving as ground truth, as those estimates are clinically meaningful but not statistically verified [27].

**Fig. 1:**
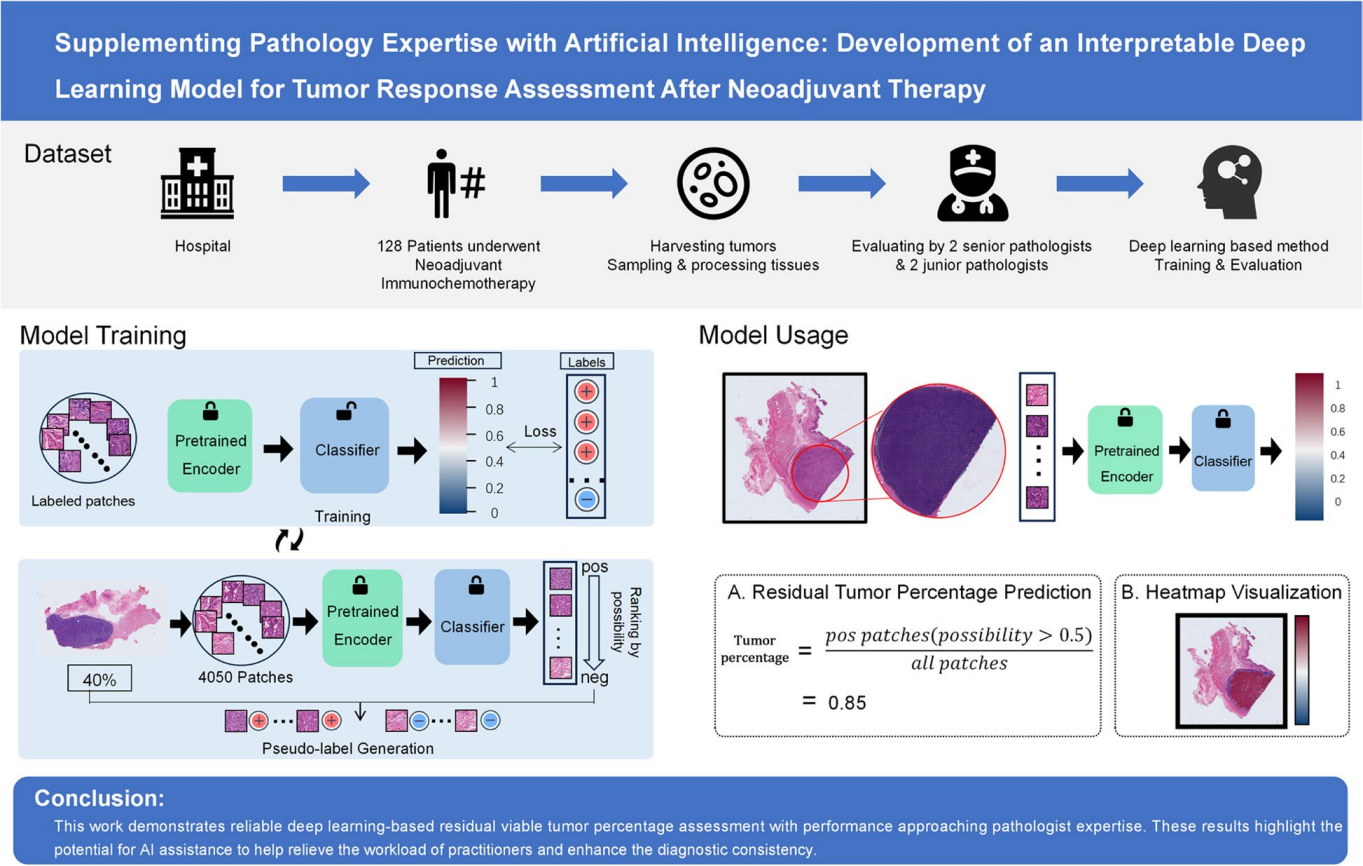
Graphical abstract of the study design and highlights

To overcome these obstacles, we developed a novel deep learning system tailored for tumor response assessment. Instead of relying only on slide-level weak supervisions, our approach uniquely incorporates both patch-level and slide-level labels through semi-supervised knowledge distillation. This model demonstrated a strongly quantitative and qualitative performance, with residual tumor percentage estimation closely paralleling the results of experienced pathologists. Across two independent test sets, our reproducible model achieved ${{\text{R}}}^{2}$ scores of 0.8437 and 0.7450 respectively for predicting pathologist-derived residual tumor percentages, significantly exceeding junior pathologists. In addition, our model generated visually interpretable heat-maps, highlighting regions of possible tumor involvement, which could serve as an assistant tool to aid pathologists, especially junior trainees, in assessing potential viable tumor areas. The visual interpretability of our approach confers a key advantage over black-box models, allowing pathologists to intuitively evaluate the rationale of model predictions. These results spotlight the potential of this novel deep learning framework to help enhance and standardize the pathological response evaluation through human-AI symbiosis. The graphical abstract of this study is shown in Fig. 1.

## Materials and Methods

### Dataset and Annotation Methods

**Fig. 2:**
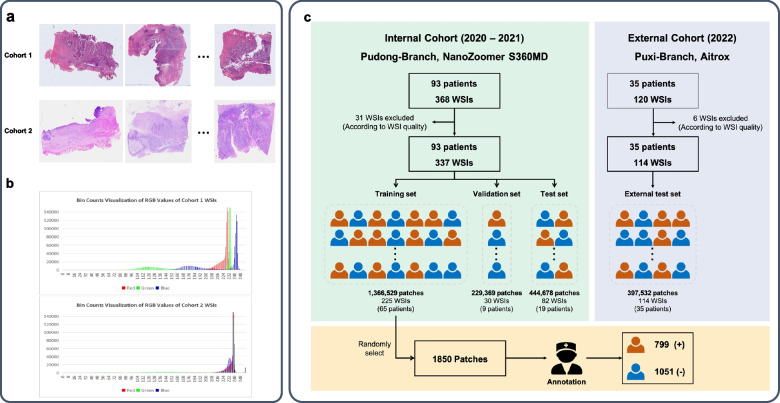
Color differences of WSIs from 2 cohorts. **a** Representative WSIs from Cohort 1 (Pudong-Branch 2020–2021) and External Cohort 2 (Puxi-Branch 2022) **b** Bin counts of red, green, blue channel values within tumor bed regions, highlighting quantitative variation between 2 cohorts. **c** Patch-level dataset generation workflow. Annotated tumor beds from pathologists were extracted from WSIs at 20 × magnification. Non-overlapping 256 × 256 pixel patches were exhaustively cropped from the tumor bed regions. The number of patches extracted per WSI ranged from 7 for small tumor beds to 21,737 for large tumor beds, depending on the annotated tumor bed size within each whole slide image

The dataset was consisted of 451 samples from two branches of Fudan University Shanghai Cancer Center (FUSCC) generated by different scanners. Cohort 1 included 337 whole slide images (WSIs) of 93 ESCC patients treated at the Pudong-Branch from 2020–2021. These WSIs were scanned using a Hamamatsu Photonics NanoZoomer S360MD Slide scanner. Cohort 2 served as an external test set, consisting of 114 WSIs from 35 patients treated at the Puxi-Branch in 2022. WSIs of Cohort 2 were generated by Digital Micro Image Analysis System from Shanghai Aitrox Technology Corporation Limited. Cohorts were Stratified by hospital branches, scanning machines, and years, given the variability in whole slide imaging equipment and other potential effects on analysis. Here we illustrated the color variation of the two cohorts by WSI examples (Fig. 2a) and bin count visualization of the RGB channel values of the tumor bed regions (Fig. 2b). In addition, WSIs from Cohort 1 were randomly split into training (*n* = 225), validation (*n* = 30) and test (*n* = 82) sets. The development of the dataset was illustrated in Fig. 2c.

**Fig. 3:**
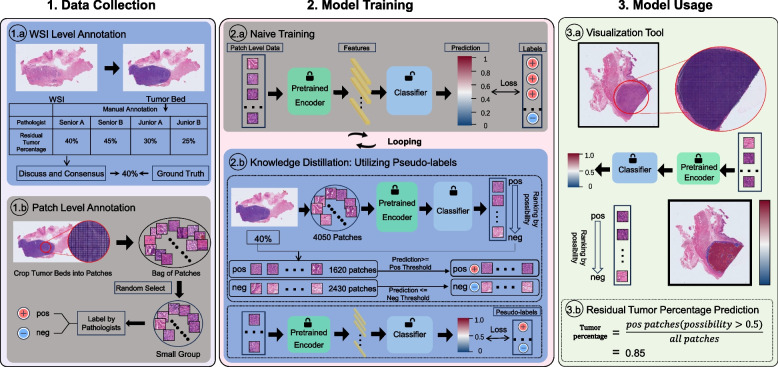
1) Data annotation protocol, comprising whole slide image (WSI)-level viable residual tumor percentage labels and patch-level binary classification of positive vs negative regions. 2) Two-phase training paradigm, first exploiting cross-entropy loss for labeled patches, followed by training with pseudo-labeled WSI patches determined by the pretrained model. 3) Exemplar clinical applications of the model for residual tumor percentage quantification and spatial mapping of potential viable regions.  shows where model weights were frozen

Pathological response assessment based on WSIs involved multiple levels of annotation, as shown in Fig. 3. Initial independent assessments were conducted by two board-certified senior expert pathologists with over 10 years of subspecialty experience, as well as two junior pathologists with around 2 years of experience. The residual viable tumor percentage was equal to the area of viable tumor divided by the area of tumor bed. And the area of the tumor bed was the sum of the viable tumor area, the necrotic area and the stromal area, totaling 100%. To derive consensus ground truth labels, the senior pathologists subsequently reevaluated each WSI jointly, reconvening to deliberate any cases of discrepancy through collaborative discussion. In other words, the ground truth was the unanimously agreed-upon result of the two senior pathologists. Notably, the assessment results of the senior pathologists achieved a strong ${{\text{R}}}^{2}$ score of 0.9202 and 0.9619 with the consensus labels, indicating robust inter-rater agreement. The resulting unified tumor percentage labels constituted the gold standard annotations for subsequent training and evaluation of the computational pathology system. When compared to the senior pathologists’ annotations, the junior pathologists achieved much lower ${{\text{R}}}^{2}$ scores of only 0.5592 and 0.5474, highlighting the discrepancy in accuracy between senior expert and junior trainee assessment. Pathological tissue sampling details were illustrated in the Additional file 1: Appendix C section.

In addition to WSI-level percentage estimates, patch-level labels were obtained to facilitate deep learning. Tumor bed regions were extracted from the WSIs and were cropped into 256 × 256 pixel patches at 20 × magnification. 1850 patches from the training set were randomly selected and manually annotated by the senior pathologists, designating each patch as positive if viable tumor cells were present, or negative if no tumor cells were visible. This patch-level labeling allowed direct supervision of tumor localization and morphology patterns at the patch scale, complementing the coarse slide-level percentage estimates.

### Establishment and Evaluation of the Deep Learning-Based System

Our pathological tumor response model was built on patch-level classification of tumor contents. Specifically, cropped patches were classified as positive or negative based the presence or absence of viable tumor cells. The proportion of positive patches within the tumor bed region provided whole-slide estimates.

A two-stage semi-supervised approach (Fig. 3) was employed to train our model for the whole slide tumor percentage estimation. Specifically, to reduce computation cost, a pretrained encoder [27] was used to extract features from the patches and then a two-layer fully connected neuron was used as a classifier.

In our two-step training, we first trained the classifier on the pathologist-labeled patches to learn the morphological patterns which associated with viable tumor cells. Then in the second step, for each training WSI, all unlabeled patches from the tumor bed were evaluated and ranked by the predicted probability of containing viable tumor. We applied the pathologist-derived slide-level percentage along with a threshold to assign pseudo-labels. Details of these two steps can be found in the Additional file 1: Appendix B.

We iteratively trained the two steps for several iterations. The final classifier generated the tumor likelihood heatmaps to visualize the predictions (Fig. 6), which can assist pathologists in evaluating areas of potential tumor involvement. To assess the slide-level residual tumor percentages, the predicted positive patches were aggregated and divided by the total tumor bed area.

### Evaluation Metrics

We evaluated model performance using both quantitative and qualitative measures. Quantitatively, we employed several complementary metrics: ${{\text{R}}}^{2}$ regression score, mean absolute error, mean squared error, and robust accuracy. Details of the evaluation metrics can be found in the Additional file 1: Appendix A section.

## Results

### The Correlation Between Pathological Response and the Benefit of Survival

A total of 128 ESCC patients from cohort 1 and cohort 2 were included. All the patients had neoadjuvant therapy followed by surgery at different branches of Fudan University Shanghai Cancer Center (FUSCC) from January 2020 to December 2022. The clinicopathological and treatment characteristics were illustrated in Table 1 in the Additional file 1: Appendix D section. All the patients received immunotherapy combined with chemotherapy. The median age of the patients was 61 in cohort 1 and 63 in cohort 2. Most of the patients were male with a smoking history. All the patients received docetaxel plus cisplatin (TP regimen) for every 21 days for 2 cycles. Regarding immune checkpoint blockade, nearly half of the patients (46.2% and 40%) of the patients received Camrelizumab and about 30% of the patients received Pembrolizumab (29% and 31.4%).

The degree of pathological response was accessed by pCR and MPR. pCR was defined as the absence of viable residual tumor and MPR was defined as less than or equal to 10% of the viable residual tumor. Among the 128 patients in cohort 1 and cohort 2, about 25% of the patients achieved pCR (32/128) while 35.2% of the patients achieved MPR (45/128). OS and PFS were defined from the time of surgical resection to death/disease progression. yTNM being a well-accepted assessment metric of post-treatment disease status was also generated for the comparison of prognostic prediction ability. Detailed information regarding patients’ yTNM stage was illustrated in Table 1 in Additional file 1: Appendix D section. (yTNM implied the features of the primary tumor(T), regional lymph nodes(N) and the presence or absence of distant metastasis(M) based on post-therapy findings).

**Fig. 4:**
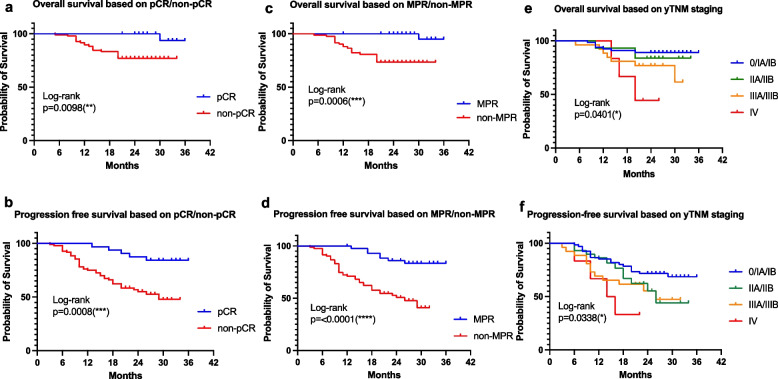
The correlation between pCR, MPR, yTNM and the benefit of survival. Both pCR and MPR were predictive of a longer OS and PFS (**a**-**d**). Regarding the well-accepted assessment metric yTNM, patients with a lower yTNM stage were also shown having a greater likelihood of obtaining better survival benefits as well (**e**–**f**). (pCR: the absence of viable residual tumor; MPR: less than or equal to 10% of the viable residual tumor. yTNM implied the features of the primary tumor(T), regional lymph nodes(N) and the presence or absence of distant metastasis(M) based on post-therapy findings; OS: overall survival; PFS: progression-free survival)

The survival curves were plotted in Fig. 4(a-f). At the time of analysis, with a 36-months follow-up, patients with pCR had a significantly better OS (Fig. 4a, *p* = 0.0098) and PFS (Fig. 4b, *p* = 0.0008). Likewise, MPR was also significantly predictive of a longer OS (Fig. 4c, *p* = 0.0006) and PFS (Fig. 4d, *p* < 0.0001). As a reference metric, patients with a lower yTNM stage were also shown having a greater likelihood to obtain better survival benefits (Fig. 4e, *p* = 0.0401) and PFS (Fig. 4f, *p* = 0.0338).

### Inter-Observer Agreement Between Pathologists

Four pathologists at different experience levels independently assessed the WSI in our dataset, which included two senior pathologists with over 10 years of specialty experience, and two junior pathologists with 2 years of experience.

Two senior pathologists achieved a high inter-observer concordance with the consensus labels, with ${{\text{R}}}^{2}$ scores of 0.9202 and 0.9619, while comparisons of junior to senior pathologists yielded markedly lower agreement, with ${{\text{R}}}^{2}$ socres of 0.5592 and 0.5474. More details of the comparison among pathologists at different level could be found in Table 2 in the Additional file 1: Appendix D section.

In all, senior pathologists exhibited robust concordance in tumor percentage estimation, providing a reliable consensus gold standard. Junior pathologists showed only fair to moderate agreement with senior raters, with a tendency for overestimation bias.

**Fig. 5:**
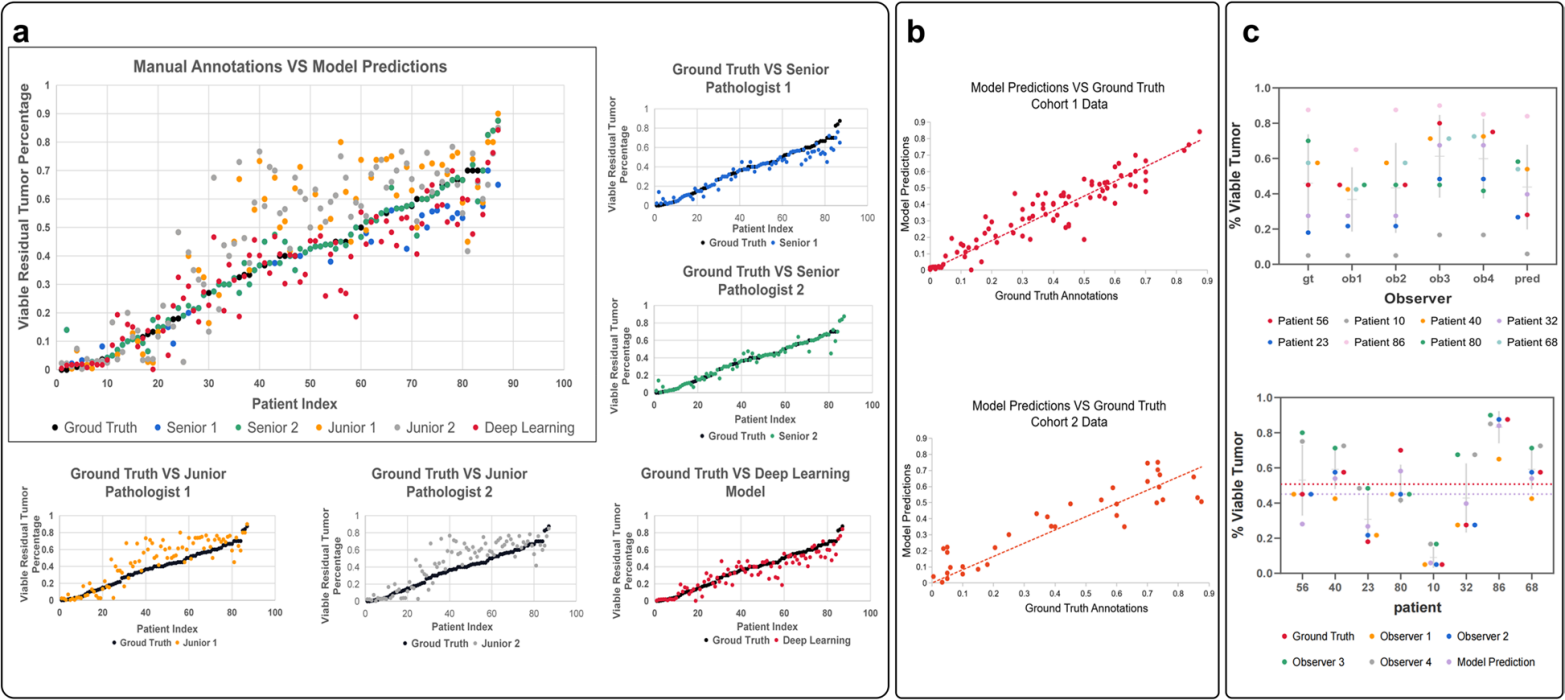
**a** Comparative assessment of residual tumor percentage by different analytical approaches versus expert consensus ground truth. **b** Assessment of model-predicted residual tumor percentages against ground truth measurements across two study cohorts. The performance remained notable on the external Cohort 2 test set, showing satisfactory generalizability of our approach to new histological specimens. **c** Scatter plots comparing viable tumor percentage predictions for example patients from human experts and the deep learning model, illustrating general concordance between automated and manual assessment methods

As shown in Fig. 5, junior pathologists tended to substantially overestimate residual tumor percentages when compared to senior pathologists. Further analysis revealed that this might have been due to the misidentification of some reactive cells, such as giant cells and histocytes, as degenerated tumor cells, as well as the failure of excluding the interstitial fibrous tissue that was interspersed among the tumors.

These results indicated that the results of different observers could be inconsistent and precise tumor response assessment might be challenging for less experienced pathologists. Hence, the exploration of computational aids is necessary, considering its reproducibility may help improve inter-observer reliability, particularly in less experienced practitioners.

### Comparison Between Manual and Dl-powered Assessment

Both quantitative and qualitative results demonstrated the efficacy of our deep learning-based assessment system for residual tumor estimation on Cohort 1. As shown in Fig. 5 and Table 3 in the Additional file 1: Appendix D section, our model achieved an ${{\text{R}}}^{2}$ of 0.8437 against consensus labels, and a ${{\text{RAcc}}}_{0.3}$ of 0.9885. This level of accuracy significantly exceeded those of junior pathologists and closely approaching those of senior experts. Thus, for the original training dataset, this deep learning model was proved capable of reliable residual tumor percentage estimations comparable to experienced human observers.

When applied to the external cohort 2 test set, the performance of this model remained notable, as shown in Fig. 5b. Specifically, this model attained an ${{\text{R}}}^{2}$ of 0.7450 and a ${{\text{RAcc}}}_{0.3}$ of 0.9428 on this external dataset, as presented in Table 3 in the Additional file 1: Appendix D section. While metrics were modestly decreased versus cohort 1, this evaluation demonstrated satisfactory generalizability of our approach to new histological specimens. By achieving reliable estimates on both related and distinct cohort images, our deep learning system substantiated its potential as a computational tool to empower accurate and consistent tumor response assessment.

### Visualization and Interpretability

**Fig. 6:**
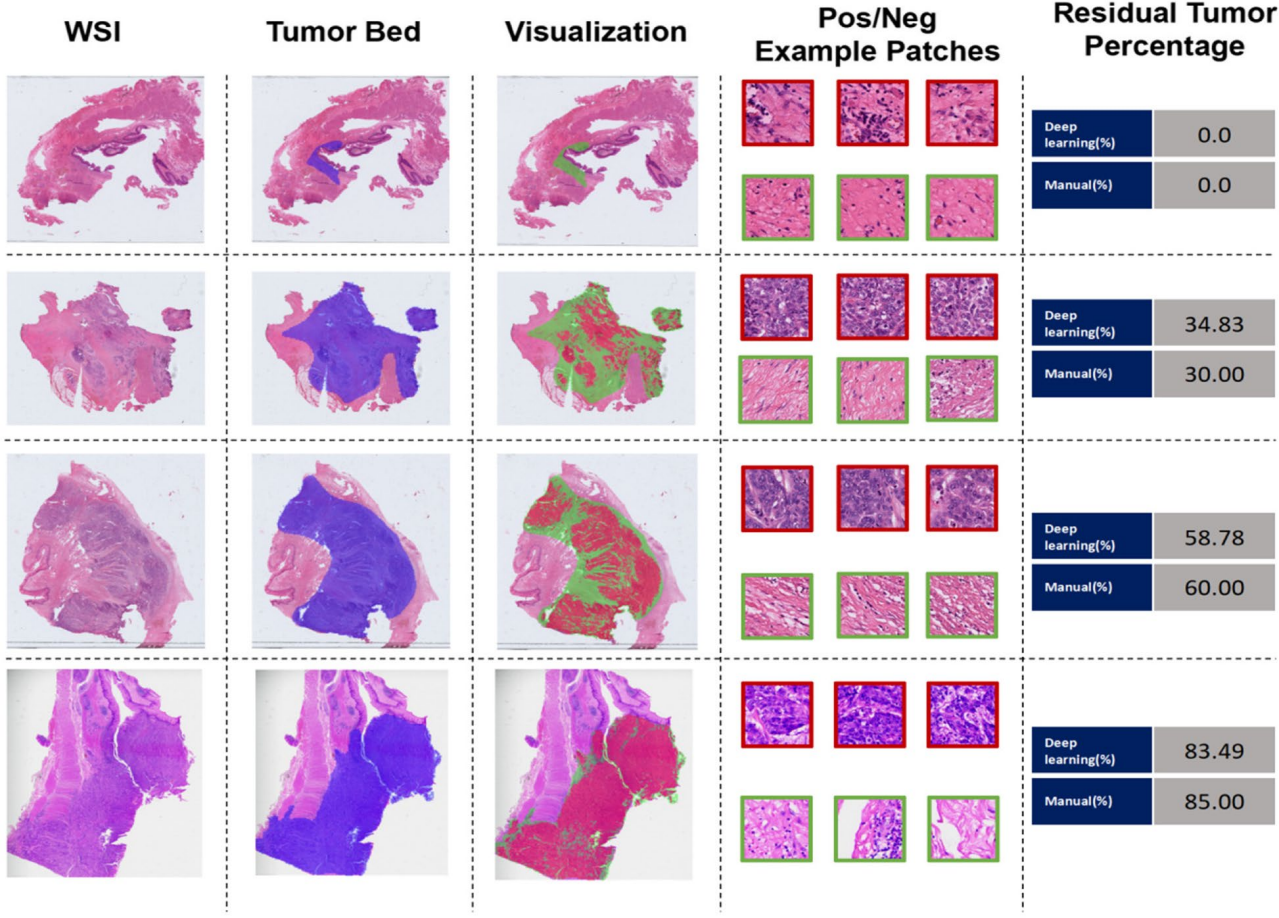
Demonstrative potential clinical application of the deep learning framework, enabling automated viable residual tumor percentage estimation and spatial mapping to augment pathological response assessment

To convey the interpretability of our model’s predictions and tumor localization capabilities, we generated various visualizations using representative test cases. Figure 6 displayed heatmaps and classified patches from four whole slide images and compared against ground truth assessments. Specifically, each row presents: 1) the whole slide image, 2) tumor bed area 3) the full-slide heatmap of estimated tumor likelihood, and 4) high and low possibilities patches. Alongside each row, we reported the predicted ground truth residual tumor percentages. This visualization demonstrated the model’s capability to localize tumor regions and provide reasonable patch-level classification, while also evaluating the slide-level residual tumor percentage. Overall, these visualization tools support the model’s reliability and interpretability for clinical application in pathological tumor assessment.

## Discussion

Pathological assessment after neoadjuvant treatment is one of the most important criterions for evaluating the effectiveness of preoperative treatment methods and usually serves as the basis for formulating post-surgical treatment plans. It is also considered a surrogate endpoint for clinical trials, aiming to shorten the time required for evaluation. Inevitably, in our study, patients achieving pathological remission (pCR, MPR) were associated with better survival benefits (OS, PFS). However, pathological assessment can be labor-intensive in high-risk areas and practitioners with varying levels of experience may have inconsistent results due to subjectivity.

This study demonstrated the potential of a deep learning-based model for identifying residual viable tumor cells and assessing whole-slide residual tumor percentage. By aggregating patch-level predictions, this model was able to generate slide-level predictions that strongly correlated with while minimally differed from expert assessments across both independent cohorts, despite inter-cohort differences in hospital branch, scanning equipment, and years.Tumor likelihood heatmaps provided a spatial localization of involved regions, serving as a valuable visualization tool, which may assist practitioners for potential tumor involvement prediction in clinical practice. These results indicated the capability of AI-assisted computational methods for reliable tumor response assessment after neoadjuvant therapy, which is crucial for personalized post-operation treatment strategy-making.

Several findings emerged from the analysis of the model output versus pathologists’ performance. First, although senior pathologists exhibited high inter-observer concordance, providing a robust gold standard for evaluation. Junior pathologists demonstrated only fair to moderate agreement with the seniors, largely due to their misidentification of the viable residual tumor content and the failure to exclude the interstitial fibrous tissue that was interspersed among the tumors. This finding mirrored some known challenges of accurate and consistent pathological response assessment, especially for less experienced practitioners, while the advantages of deep learning assistance may largely compensate these weaknesses.

Second, the accuracy of this DL-powered model surpassed that of junior pathologists, while closely approaching expert-level performance. Quantitative metrics and spatial heatmaps highlighted the capacities for tumor localization and residual tumor percentage estimation, rivaling specialty pathologists. As an adjunct computational aid, such systems may reduce the inconsistency and help standardize the pathological response assessments, particularly among less experienced practitioners.

Third, with the increasing popularity of preoperative neoadjuvant therapy, pathological response assessment can be labor-intensive and time consuming. With further enhancement of the AI-model, pathologists may only need to review and make adjustments on the estimations generated by the AI-model, which could greatly reduce the workload and shorten the time for patients to receive the results.

Nevertheless, this system still has some limitations. First of all, the performance of this model still falls short of experienced pathologists, indicating room for improvement, which might be enhanced through training refinements and annotated training data scale. Second, since this was a pilot model, comparisons were made using tissue sample sets from a single cancer type. Broader validation across heterogeneous samples, stains, and tumor varieties is warranted. Finally, manual delineation of tumor beds is still required in this model, which is expected to be automated in the future work.

In summary, this work demonstrates a reliable deep learning-based model which is able to generate pathological response assessment after neoadjuvant therapy with performance approaching experienced pathologists. This finding indicates the potential of AI assistance to help relieve the workload of practitioners and enhance the diagnostic consistency. Following further validation, refinement, and automation of tumor bed segmentation, our deep learning-based model and accompanying visualization tool may serve as valuable supplements that empower the pathologists by improving diagnostic consistency and accuracy, particularly assisting junior pathologists in pathological response assessments.

### Supplementary Information

**Additional file 1. **Appendices A-D.

## Data Availability

No datasets were generated or analysed during the current study.
