# Supplementary material for: Development of an Interpretable Deep Learning Model for Pathological Tumor Response Assessment After Neoadjuvant Therapy
Source: Biol Proced Online. 2024 Apr 17;26:10. doi: 10.1186/s12575-024-00234-5 (PMC11022344; doi:10.1186/s12575-024-00234-5)
Supplement: Supplementary file 1 — Additional file 1. Appendices A-D. [file 12575_2024_234_MOESM1_ESM.docx]

***Appendix A: Details of the evaluation metrics***

To generate slide-level percentage estimates, predicted positive patches were aggregated and divided by the total tumor bed area. The coefficient of determination ($R^{2}$) score compared these predictions to the consensus ground truth labels from pathologists. It indicated the goodness of fit, with 1.0 meaning perfect correlation and 0 indicating performance equal to simply predicting the mean. We also used mean absolute error and mean squared error, which were commonly used in regression tasks.

Since the consensus labels represent rough visual estimates, we also calculate robust accuracy:

$$\mathrm{RAcc}_{\varepsilon}= \sum I(|\hat{y_{i}}-y_{i}| <\varepsilon) / N$$

Where $\hat{y_{i}}$ and $y_{i}$ are the predicted and ground-truth percentages for slide $i,\varepsilon$is the acceptable error margin, and$I(\cdot)$is an indicator function evaluating to 1 when the prediction is within this margin. These metric measures accuracy allowing for small deviations from the imprecise gold standard labels.

***Appendix B: Details of the training steps***

This section details the process of our two-stage semi-supervised approach to pseudo-label patches utilizations for training an interpretable deep learning model.

In the first stage, a naive supervised training utilized pathologist-labeled patches to learn tumor morphology via the classifier. Then in the second stage, unlabeled patches from each WSI underwent probability-based ranking by the model from stage one. Based on the pathologist-derived slide-level percentage and confidence thresholds, we assigned pseudo-labels to the unlabeled patches.

For example, if the slide was annotated as having 90% viable tumor cellularity, the top 90% of patches by predicted probability were pseudo-labeled as tumor-positive, and the bottom 10% were pseudo-labeled as tumor-negative. To discard ambiguous patches, we set confidence thresholds for the model - 0.9 for positive predictions and 0.1 for negative predictions. Any positive patches with the probability <0.9 or negative patches with the probability > 0.1 were excluded from the pseudo-labeled data. This knowledge distillation process generated patch-level labels with the slide-level assessment while discarding patches where the classifier was less certain. The classifier was then trained on both the pathologist-labeled patches and model-pseudo-labeled patches. We iteratively trained the two steps for several iterations to get the final stable classifier.

***Appendix C：Pathological tissue sampling strategy***

Tissue sampling and processing followed standard guidelines of IASLC Recommendations. Surgical specimens underwent fixation in 10% neutral buffered formalin for 6-48 hours. Sampling was conducted as follows: 1) For tumors ≤ 3cm, entire tumors were harvested. 2) For tumors ≥ 3cm, a minimum of 50% of the tumors were harvested by taking approximately 0.5cm cross-sections along the maximum tumor dimension. 3) Any tumors of any size with suspected pathological complete response underwent complete sampling were all harvested.

***Appendix D: Tables of information***

Table 1. Clinicopathological and treatment characteristics of the 128 patients who underwent neoadjuvant immunochemotherapy in two branches of FUSCC (Cohort 1& Cohort 2) from 2020- 2022.

| Characteristics | Cohort 1(N=93) Cohort 2(N=35) |
| --- | --- |
| Age (year)* | 47-65 (61) 50-77 (65) |
| Gender |  |
| Male | 84 (90.4%) 33 (94.3%) |
| Female | 9 (9.6%) 2 (5.7%) |
| ^*^BMI | 23 ± 3 23 ± 3 |
| †Smoking  Yes  No | 73(78.5%) 26 (74.2%)  20 (21.5%) 9 (25.8%) |
| Site of primary tumor |  |
| Upper | 12 (12.9%) 4 (11.4%) |
| Middle | 47 (50.5%) 16 (45.7%) |
| Lower | 34 (36.6%) 15 (42.9%) |
| Immune checkpoint inhibitors |  |
| Camrelizumab | 43 (46.2%) 14 (40%) |
| Prembrolizumab | 27 (29%) 11 (31.4%) |
| Tislelizumab | 14 (15.1%) 8 (22.9%) |
| Sintilimab  ^#^Postsurgical stage(yTNM)  0/IA/IB  IIA/IIB  IIIA/IIIB  IV | 9 (9.7%) 2 (5.7%)  47 (50.5%) 14 (40%)  22 (23.7%) 13 (37.1%)  19 (20.4%) 7 (20%)  5 (5.4%) 1 (2.9%) |
| *Data is shown as mean ± standard error.  †At least ten cigarettes per week for more than 6 months, including former and current smoker.  ^#^ yTNM implies the features of the primary tumor(T), regional lymph nodes(N) and the presence or absence of distant metastasis(M) based on post-therapy findings(y). | |

Table 2. Performance comparison of residual tumor percentage estimation by human experts and deep learning model on Cohort 1.

|  |  | $R^{2}$ ↑ | ${RAcc}_{0.1}$ ↑ | ${RAcc}_{0.3}$ ↑ | MAE ↓ | MSE ↓ |
| --- | --- | --- | --- | --- | --- | --- |
| Senior Pathologist | 1 | 0.9202 | 0.8506 | 1.0000 | 0.0370 | 0.0041 |
|  | 2 | 0.9619 | 0.9425 | 1.0000 | 0.0196 | 0.0020 |
| Junior Pathologist | 1 | 0.5592 | 0.5287 | 0.9080 | 0.1159 | 0.0229 |
|  | 2 | 0.5474 | 0.5287 | 0.9310 | 0.1178 | 0.0235 |
| Deep Learning | 1 | 0.8437±0.0071 | 0.7586±0.0199 | 0.9885±0.0000 | 0.0640±0.0015 | 0.0078±0.0015 |

Table 3. Quantitative performance metrics of the deep learning architecture for viable residual tumor percentage prediction on both study cohorts.

| Dataset | $R^{2}$ ↑ | ${RAcc}_{0.1}$ ↑ | ${RAcc}_{0.3}$ ↑ | MAE ↓ | MSE ↓ |
| --- | --- | --- | --- | --- | --- |
| Cohort 1 | 0.8437±0.0071 | 0.7586±0.0199 | 0.9885±0.0000 | 0.0640±0.0015 | 0.0078±0.0015 |
| Cohort 2 | 0.7450±0.0289 | 0.6571±0.0000 | 0.9428±0.0000 | 0.1044±0.0040 | 0.0223±0.0025 |
